# Development of stem cell-based therapy for Parkinson’s disease

**DOI:** 10.1186/s40035-015-0039-8

**Published:** 2015-09-03

**Authors:** Fabin Han, Deborah Baremberg, Junyu Gao, Jing Duan, Xianjie Lu, Nan Zhang, Qingfa Chen

**Affiliations:** Centre for Stem Cells and Regenerative Medicine, The Liaocheng People’s Hospital/Affiliated Liaocheng Hospital, Taishan Medical University, Shandong, 252000 China

**Keywords:** Parkinson’s disease, Dopamine neuron, Neural stem cell, Human embryonic stem cells, Induced pluripotent stem cell, Induced dopamine neuron

## Abstract

Parkinson’s disease (PD) is one of the most common neurodegenerative disorders of aging, characterized by the degeneration of dopamine neurons (DA neurons) in the substantial nigra, leading to the advent of both motor symptoms and non-motor symptoms. Current treatments include electrical stimulation of the affected brain areas and dopamine replacement therapy. Even though both categories are effective in treating PD patients, the disease progression cannot be stopped. The research advance into cell therapies provides exciting potential for the treatment of PD. Current cell sources include neural stem cells (NSCs) from fetal brain tissues, human embryonic stem cells (hESCs), induced pluripotent stem cells (iPSCs) and directly induced dopamine neurons (iDA neurons). Here, we evaluate the research progress in different cell sources with a focus on using iPSCs as a valuable source and propose key challenges for developing cells suitable for large-scale clinical applications in the treatment of PD.

## Introduction

Parkinson’s disease (PD) is one of the most common neurodegenerative disorders of aging, affecting about 1 % of the population aged 60 years and older and 3–5 % of the population above the age of 85 [1].

Clinically, patients with PD are characterized with both motor and non-motor symptoms. Motor symptoms, being far more noticeable, have typically been used in clinical diagnosis. The various disruptions in motor control include muscle rigidity, resting tremors, bradykinesia (slowness of movement), and postural instability, and typically appear when 60–80 % of dopamine (DA) neurons in the substantia nigra are degenerated [2]. The reliable identification of non-motor symptoms is important as many non-motor symptoms, including depression, cognitive dysfuction, pain, and sleep disorders, precede the motor dysfunctions; not only is management of these symptoms important for quality of life, but early diagnosis of PD could also be key for effective treatment [3].

Because DA neurons degenerate to cause a drop in dopamine release, current treatments for PD include dopamine replacement drugs such as levodopa to increase dopamine levels, dopamine inhibitor carbidopa to reduce dopamine degradation in the peripheral blood [4, 5], and deep brain stimulation (DBS) to the nucleus subthalamicus [6]. Even though dopamine replacement drugs and DBS are effective in improving the symptoms of the patients, they cannot stop the disease progression. Moreover, current medications can cause the development of dyskinesia (involuntary muscle movements), effectively “overshooting” the clinical symptoms of PD. Recent research progress has provided treatment potential through replacing lost DA neurons using neural stem cells (NSCs) or fully differentiated DA neurons from fetal brain tissue, embryonic stem cells (ESCs), mesenchymal stem cells (MSCs) sourced from adults or fetuses, and induced pluripotent stem cells (iPSCs) reprogrammed from patients’ somatic fibroblasts or blood cells. Much work has been done to adapt cells from various sources to potential clinical applications to improve treatments for neurodegenerative diseases including PD [7, 8].

### Etiology and pathological mechanisms

The causes of PD can be characterized as genetically susceptible genes and environmental toxic factors such as the pesticide rotenone and heavy metal manganese, which implicate oxidative damage and mitochondrial impairment leading to degeneration of dopamine neurons in PD [9–13]. The majority of PD cases are sporadic or idiopathic with unknown aetiology (80–90 % of PD cases), but a minority of cases (with estimates ranging from 10 to 20 % of PD cases) are familial and can be linked to a particular monogenic mutation or associated to PD related genes. The twin studies have suggested that this finding can be explained by the fact that genetic factors do not play a major role in causing typical PD, particularly with regards to disease incidence after 50 years of age [14]. They suggested that genetic factors are only an important factor when the disease begins at or before the age of 50, a relatively rare occurrence. The most well-characterized mutation loci for early-onset autosomal recessive PD are *PARK2*(*Parkin*) , *PINK1*, and *PARK7*, *ATP13A2* whereas those for the autosomal dominant form of PD are *SCNA* and *LRRK2*. The susceptible genes associated with PD are *Tau, Nurr1 and GBA. SCNA*, which codes for alpha-synuclein, has been particularly well-studied; triplication of the locus has been associated with an aggressive form of PD that advances into cognitive impairment [15, 16]. There is ongoing debate as to the exact balance between the genetic and environmental factors mainly because the accuracy of clinical diagnosis of idiopathic PD has been disputed [17]. Some other reasons of the difficulties associated with assessing the disease etiology may be the result of inconsistent diagnostic criteria among heterogeneous populations of PD patients for study.

Pathologically, PD is involved in the degeneration and loss of dopamine (DA) neurons located in the substantia nigra of the the midbrain [18]. These DA neurons project to the basal ganglia (the striatum), which is heavily involved in motor control and function [19]. The loss of DA neurons is accompanied by lewy bodies and lewy neurites, which are mainly formed by insoluble aggregates of alpha-synuclein (coded by *SCNA*) and Tau protein and might hamper the survival and dendritic development of newborn neurons [20, 21]. The spread of lewy bodies in the brain causes motor symptoms accompanied by an intensification of the disease, including cognitive impairment that encompasses hallucinations, dementia, and speech difficulties [22, 23].

### Stem cell sources for the treatment of PD

**Fig. 1:**
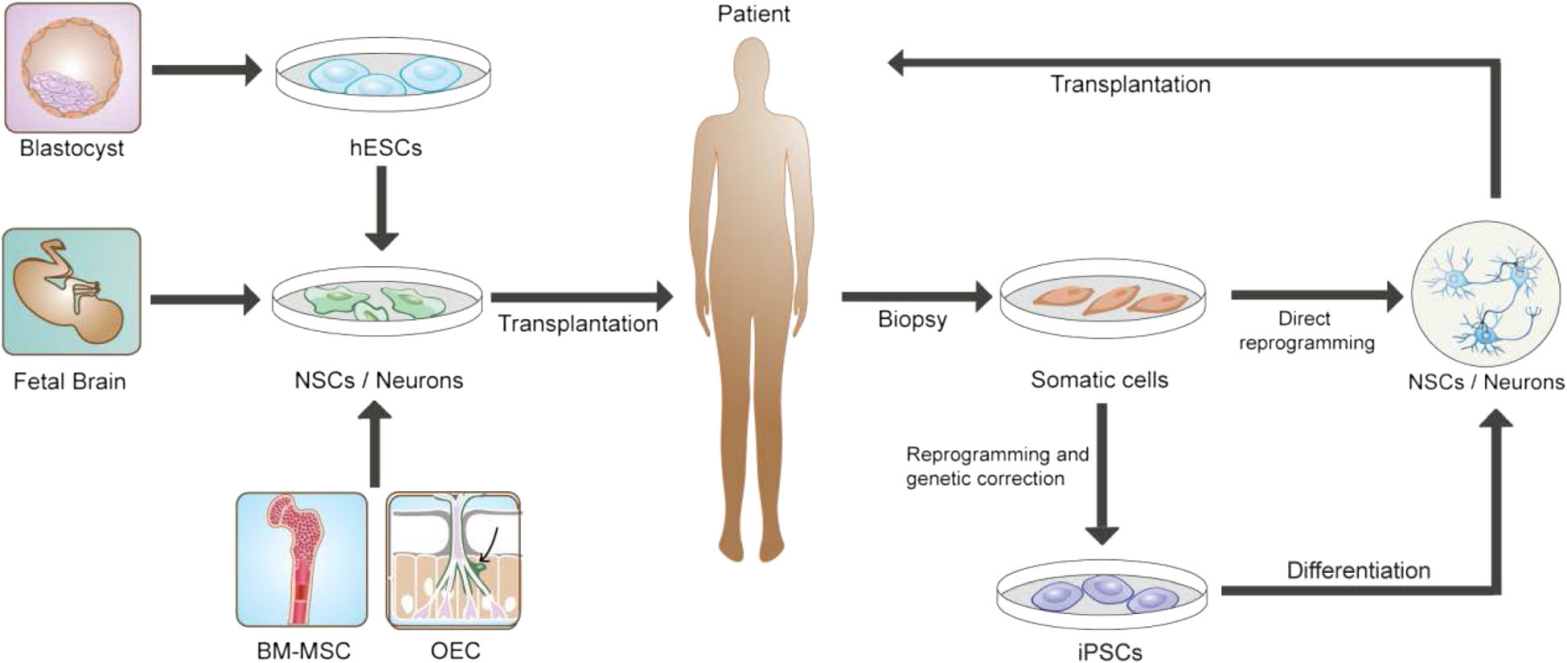
Different stem cell sources for the treatment of PD

Several stem cell sources for the treatment of PD have been studied in the past decades and summarized in Fig. 1. Some studies used adult bone marrow-derived mesenchymal stem cells (BM-MSC) and olfactory ensheathing cells (OEC) [24–26], but these cells have limited ability to differentiate to dopamine neurons. Currently neural stem cells (NSCs) and dopamine neurons from fetal brain tissue, embryonic stem cells (ESCs), induced pluripotent stem cells (iPSCs) and directly induced dopamine neurons (iDA neuron) reprogrammed from autologous somatic cells have been widely studied to move these cells into bedside [27–29].

#### NSCs and dopamine neurons from fetal brain tissue

Neural stem cells (NSCs) were first reported in 1965 [30] and were described as granule cells with a high rate of proliferative activity in the cortex of brains. As a multipotent stem cell population, NSCs have neural potential and can differentiate into neurons, astrocytes and oligodendrocytes. NSCs can be also isolated from the other regions of fetal brains or from the hippocampus and subventricular zone (SVZ) of the adult mammalian brain, the areas where neurogenesis continues throughout the animal’s lifespan [31, 32]. Since initial discoveries of NSCs, there have been many advances in the isolation, expansion, and differentiation of NSCs [33, 34]. Mouse and human NSCs transplanted into the rat brain migrate and differentiate in a site-specific manner. Moreover, Nishino et al. reported that these NSCs appear to differentiate preferentially into DA neurons in PD model rats with depleted host DA levels [35].

NSCs can be also induced to differentiate into specific neural cells in vitro prior to transplantation. Some homeodomain transcription proteins were isolated and selectively expressed in DA neural progenitors in the ventral midbrain, and it was found that *Lmx1a* and *Msx1* function as lineage determinants triggering generation of DA neurons with midbrain identity. These factors were shown to initiate the differentiation of neural progenitor cells in chick embryos into midbrain DA neurons. These findings suggested that *Lmx1a* and *Msx1 *play important roles in the specification and maturation of DA neurons. NSCs were differentiated into DA neurons via a five-step protocol similar to the culture conditions used to differentiate ESCs demonstrated the morphological characteristics of forebrain DA neurons. Kim et al. reported that overexpressing the transcription factor *ASCL1* was able to regain neurogenesis from human neural progenitor cells and produced larger neurons with more neurites [36]. The identification of *NURR1* mutation in PD patient suggested that *NURR1* plays regulatory role in the development of DA neurons [37]. Forcing overexpression of *Nurr1* was found to enhance the ability of mouse NSCs to differentiate into DA neurons and survive in vivo in PD rat models [38].

Animal studies showed that rodent and human fetal brain dopamine neurons transplanted to the midbrain of the 6-OHDA-lesioned rats survived well in the host brains and improved the motor defects of the PD rats [39]. Even though some studies reported limited recovery after transplanting fetal substantia nigra-derived cells into rat PD models, most found very promising results [40, 41]. Redmond et al. reported fetal ventral mesencephalic (VM) tissue transplanted to the 1-methyl-4-phenyl-1,2,3,6-tetrahydropyridine (MPTP)-lesioned African Green Monkeys (AFG) survived well in the host brains, and all animals showed significant behavioral improvement in primate model of PD by 9 months post-transplantation [42]. Based on the animal studies, the first clinical trials began in Sweden in the late 1980s to transplant fetal dopaminergic neurons or tissue to PD patients in placebo-controlled protocols [43]. Subsequently, the clinical assessment protocols were modified to use the quantitative measurements of motor function, and several clinical trials were conducted to transplant human fetal brain-derived dopamine neurons to PD patients. In terms of behavioural and histological improvements, significant effects were found in these small-case studies [44, 45]. Freed et al. performed double-blind, sham surgery-controlled study by selecting 40 patients with mean PD duration of 14 years and randomly dividing the patients into two groups of 20 patients each. The transplantation group was injected with fetal brain neural cells bilaterally whereas the control group received sham surgery. All the patients were evaluated at one year after transplantation based on the Unified Parkinson’s Disease Rating Scale (UPDRS). As a result, significant improvements were found for younger PD patients at the age of 60 years old and younger whereas no significant improvements were found in older patients compared to the control group, implying that the therapeutic efficacy varied in certain subpopulations [45]. In general, clinical trials have had extremely variable functional outcome, though solid improvements need to be further determined by clinical and imaging evaluations [46, 47]. Olanow et al. performed another double-blind controlled clinical trial with 34 severe PD patients for two years after transplantation. Patients were randomly received bilateral transplantation of fetal nigral neural cells as transplantation group or sham surgery as control group. Overall no significant therapeutic effects were in transplantation group versus the control group even though robust survival of dopamine neurons was observed at postmortem examination [48]. Interestingly in another double-blind study, 33 patients who were transplanted with fetal brain dopamine neurons were followed for 2 years and 15 of these patients were followed for 2 more additional years, a significant clinical improvement in UPDRS motor ratings and increase in putamen uptake on (18)F-fluorodopa ((18)F-FDOPA) PET indicated the viability of the fetal brain grafts in PD patients over the 4 year course of the study [49].

However, fetal brain tissue transplantation did not escape the side effect of dyskinesia, prevalent in more traditional levodopa treatments for PD. Olanow et al. found that 56 % of patients into which fetal mesencephalic tissue was transplanted developed persistent dyskinesia after overnight withdrawal of dopaminergic medication [48] – far more than 15 % of patients experiencing dyskinesia Freed et al. reported [45]. Though its exact prevalence may be contested, the recurrence of dyskinesia following neural transplantation has been well-documented [46, 50]. There is evidence that grafts containing serotonin neurons are more likely to have this detrimental effect and that dyskinesia may therefore be alleviated by ensuring a homogeneous cell population in transplantation [51, 52]. In order to know the long-term results with fetal brain cell transplantation, three individual clinical trials were studied. One study found that transplanted fetal midbrain DA neurons survived without pathology after up to 14 years, suggesting the safety and feasibility of transplanted fetal brain cells for the treatment of PD [53], other two studies found that alpha-synuclein-positive lewy bodies eventually spread to the transplanted DA neurons in PD patients after 14 or 16 years of transplantation [54, 55]. These pathological changes suggest that PD can be an ongoing process.

The discrepancy may be the result of the difference between genetically and environmentally caused PD – a case of PD caused by genetic mutations would be an ongoing process, whereas a case of PD caused by environmental factors might be halted by the infusion of healthy cells. Therefore, it cannot be conclusively stated that DA neuron engraftment is a universally permanent treatment for PD; follow-up implantations may be required for optimal effectiveness. However, fetal midbrain cell transplantation did provide PD patients, on average, symptomatic improvements when compared to control groups, but it is not a recommendable therapy for PD unless significant improvements are made and issues regarding to consistency of improvements, recurrence of dyskinesia, and eventual spread of pathology are overcome. There is also, like all other allogeneic treatments, a risk of graft rejection – the fact that the midbrain tissue with which a patient is being treated has been sourced from a genetically distinct individual, causing immunogenic responses that must be repressed in the study [56, 57].

Moreover, the use of fetal primary tissue for PD treatment is not scalable, given the procurement difficulty and ethical concerns behind the use of NSCs from fetal brain tissues. Overall, the clinical trials with NSCs of fetal brains showed some improvements of symptoms and the survival of the transplanted cells in PD patients, but some results are controversial because of the limited cases or diversities of the PD patients [47]. Some of the clinical trials with fetal brain- derived NSCs or dopamine neurons are summarized in Table 1.

**Table 1:** Summary of the clinical trials using fetal brain cells for treatment of PD

No. of patients with cell transplantation	Follow-up Time	Symptom improvement	PET DA neuron survival	Side effect of Dyskinesia	Pathological lewy body	References And publication year
1	12 months	1/1	Yes	No	Not available	[43]
6	10–72 months	4/6	Yes	No	Not available	[77]
5	18–24 months	2/5	Yes	No	Not available	[36]
20/40	3 years	17/20	Yes	No	Not available	[45]
23/34	24 months	6/23	Yes	56 % dyskinesia	Not available	[48]
2	8 years	2/2	Yes	50 % dyskinesia	Not available	[7]
5	9–14 years	Not available	Yes	Not available	Not available	[42]
1	14 years	1/1	Yes	Dyskinesia	Lewy body	[55]
2	11–16 years	Not available	Yes	Not available	Lewy body	[54]
33	2–4 years	45 %	Yes	Not available	Not available	[8]
3	13–16 years	Yes	Yes	Not available	Not available	[8]
2	18 and 15 years	2/2	Yes	Not available	Not available	[24]

Note: 20/40 and 23/34 indicates that 20 of 40 patients and 23 of 34 patients are in the cell transplantation group and the other patients are in the control group

In order to further address the therapeutic effects of the transplanted NSCs for patients, a new multicenter and collaborative study of the European Union (TRANSEURO) was formed in 2010 to make new guidelines for clinical trials of fetal brain-derived cell therapy in PD. These include careful selection of patients: aged 30–68 at the time of inclusion, showing a good response to levodopa; early in the course of their disease (disease duration 2–10 years); systematic evaluation of cell preparation location of transplantation; clinical assessment standards; numbers of patients and; immunosuppression after transplantation and follow-up time. This study has completed the new clinical trial for more than 100 PD patients and results are in the analysis [58, 59]. A significant clinical outcome was recently reported in two PD patients who were transplanted with fetal ventral mesencephalic cells and were followed up to 15 and 18 years post-transplantation. The motor improvement was observed in the first year and continued to 18 years after transplantation with discontinued levo-dopa replacement therapy [60].

#### Human embryonic stem cells (hESCs)

Embryonic stem cells (ESCs) are pluripotent, self-renewing, and isolated from the inner cell mass of the pre-implantation blastocysts [61]. ESCs can therefore be differentiated into any kind of tissue cells, including neural stem cells (NSCs), neurons and DA neurons. Mouse embryonic stem cells-derived NSCs, or fully differentiated neurons and dopamine neurons have been shown to have neuroprotective effects for the treatment of PD [62–64].

Human embryonic stem cells (hESCs) were first isolated by culturing inner cell mass cells with feeder cells of mouse embryonic fibroblasts (MEFs) [65]. In the past two decades, strategies have been developed to direct the hESC differentiation into the neural stem cells and neurons, in particular dopamine neurons for PD. Studies have shown the differentiation of hESCs into midbrain dopamine (DA) neurons by the application of specific patterning molecules that regulate midbrain development in vivo [66, 67]. Transplantation of hESCs-derived neural precursor cells to the PD rats showed that grafted cells differentiated DA neurons in vivo and development of protocol for producing more DA neurons in vitro is required [68]. Moreover, Yan et al. developed protocols for generating specifically midbrain-like DA neurons from hESC-derived neuroepithelial cells by applying growth factors SHH and FGF8 in a specific sequence [69]. Their study suggested that early exposure to growth factor FGF8 and SHH instructs early precursors to adopt a region identity leading to differentiation of midbrain neuroepithelial cells. These hESC-derived dopamine neurons were able to improve the locomotive deficits of PD rat models, provided that grafted hESC-derived dopamine neurons functioned in vivo [70]. In order to increase the efficiency of DA production from pluripotent stem cells, Chamber et al. developed a protocol by inhibiting SMAD signalling to enhance proliferation and survival of midbrain DA neurons from hESCs [71]. They reported that addition of Noggin and SB431542 for inhibiting SMAD signalling induces complete neural conversion of >80 % of hESCs under adherent culture conditions. Fasano et al. have reported that neurons in developmental default towards anterior regionalization, but may be shifted towards a midbrain- like identity by FGF8 or Wnt1 treatment [72]. In order to further improve complete conversion of hESCs to the dopamine neurons and decrease the teratoma potential in vivo, the same group developed a floor-plate-based method for generating hESCs-derived DA neurons in a differentiation medium containing activators of sonic hedgehog (SHH) and canonical WNT signalling in vitro. They found that these DA neurons efficiently grew for more than 18 weeks and restored the amphetamine-induced rotation dysfunctions in vivo after being transplanted into 6-OHDA-lesioned rats and MPTP-lesioned rhesus monkeys [73]. Muramatsu et al. implanted NSCs derived from cynomolgus ES cells unilaterally in the putamen of neurotoxin- lesioned cynomolgus monkeys. They found that transplantation of NSCs derived from cynomolgus monkey ES cells can restore DA function in a primate model of PD [74]. Another group reported that using lentiviral vectors to express the key DA neuron-regulating gene, *LMX1A*, in hESCs produced ventral midbrain DA neurons of the A9 subtype which account for more than 60 % of all neurons generated from *LMX1A*-transfected hESCs [75]. To determine the functional properties of hESC-derived DA neurons in vivo, hESC-derived midbrain dopamine neurons and fetal brain DA neurons were engrafted into rat models of PD. MRI and PET imaging analysis showed that grafted hESC-DA neurons survived, projected long neural branches, and played similar functions to improve the locomotive deficits of PD rats as fetal brain DA neurons, providing further preclinical evidence of hESC-derived dopamine neurons for treatment of PD [76].

The major concerns to use stromal cell as feeder cells for culturing hESCs-derived cells for clinical purpose are that hESCs-derived cells contain some rodent cells and may increase the risk of immune rejections. To overcome this problem, some studies developed feeder-free culture system to use matrigels to replace the feeder cells [36, 77]. Schulz et al. moved towards clinical applicability by generating the neurons in a serum-free suspension system [78]. Vazin et al. were successful in replacing the PA6 stromal cells with growth factors SDF-1, PTN, IGF2, and EFNB1, which induced the differentiation of hESCs directly into TH-positive DA neurons [79]. Growth factors SHH and FGF8 were reported to substitute for PA6 stromal cells in generating DA cells after an initial induction step of differentiating hESCs into NSCs. They endeavoured to develop a scalable process applicable to the clinic and easily brought to Good Manufacturing Protocol (GMP) standards. Their culture protocols did not involve serum, but they made the important discovery that cells could be stored at each of the intermediate stages in their four-step process (propagation of ESC → generation of neural stem cells (NSC) → induction of dopaminergic precursors maturation of dopaminergic neurons) without loss of functional ability, allowing cells to be transplanted at an appropriate time point in neural development [80].

Although hESCs can be efficiently differentiated into a large amount of DA neurons in vitro and showed solid functions to restore the motor dysfunctions in PD animal models, including mice, rats and non-human primates, clinical trials have not been performed for treating PD patients. The main problems with hESCs are: i) the phenotypic stability of hESC-derived dopamine neurons after transplantation, and ii) the worry about residual undifferentiated hESCs within the large numbers of cells that need to be injected for human therapy. The residual undifferentiated hESCs might indeed lead to tumor formation even if this is not observed anymore in rodent experiments. In addition, some ethical concerns and problems of immune rejection also limited the clinical applications of hESCs.

#### Induced pluripotent stem cells (iPSCs)

Earlier studies showed that differentiated somatic cells could be reprogrammed to an undifferentiated state using somatic cell nuclear transfer (SCNT). SCNT technology made the cloned lambs and cows available [81–83]. But generating patient-specific cells using this technique has not yet occurred [84, 85]. The successful generation of mouse iPSCs was first reported in Yamanaka lab in 2006 by lentiviral expression of four transcription factors: *Oct3/4, Sox2, c-Myc, and Klf4 *in mouse embryonic fibroblasts [86]. Soon afterwards, the Yamanaka lab, as well as other labs, used the human orthologs of these four transcription factors (*OCT4, SOX2, c-MYC, KLF4*), or *OCT4, SOX2, NANOG and LIN28*, to generate human iPSCs and patient- specific iPSCs with different diseases, including PD [86–88]. The implication of *Oct3/4* and *Sox2* was unsurprising as previous research defining their essential role in the propagation of undifferentiated ESCs in culture [89]. The roles of *Klf4* and *c-Myc* were promoting reprogramming of somatic cells. Later studies indeed showed that* Oct3/4* and *Sox2* appear to be the only genes indispensable in generating iPSCs [90] whereas *Klf4* and *c-Myc* are disposable [91]. iPSCs share most of their characteristics with ESCs; they are pluripotent and self-renew indefinitely. However, they are generated via reprogramming the already-differentiated somatic cells of an organism back to their embryonic-like pluripotent state. Overcoming the problems associated with fetal NSCs and hESCs, iPSCs generated from patients will have wide applications for exploring the molecular mechanisms and cell-based therapy of neurodegenerative diseases [28, 86, 88, 92].

One of the major advantages of iPSCs over other cell types in clinical applications is that iPSCs can be generated from the cells of the individual being treated; the cultured cells will be autologous. This key trait of iPSCs theoretically minimizes the risk of rejection and enhances their integration into the brain tissues of patients with PD. Moreover, the ethical issues of using aborted fetuses as a cell source are circumvented. Once reprogrammed into iPSC state, the iPSCs can be systematically exposed to specific factors that direct the cells to differentiate into a specific lineage (such as NSCs or DA neurons) [93, 94]. Much research has been done to improve the generation, differentiation, and potential clinical applications of iPSCs, with particular efforts made to bring these therapeutic cells to GMP (Good Manufacturing practice) standards such that they can be translated to the clinic for treatment of neurodegenerative diseases such as PD [95]. iPSCs have also been used in drug screening and discovery and as a disease model to study the molecular mechanisms of the disease [96].

To determine the clinical potential of iPSCs-derived cells, the therapeutic effects of mouse iPSCs were studied by transplanting the iPSCs into rat brains [97]. They found that grafted iPSCs matured into midbrain-like dopamine neurons, resulting in behavioural improvements in rat PD models. In other studies, human iPSCs-derived neurons or neural stem cells showed therapeutic effects in rat and monkey PD models [9, 98–100]. Even though many studies indicated the iPSCs induced improvement of motor function in animal PD models, no clinical trials have been reported using human iPSCs for the treatment of PD patients. Because the integration of viral vectors and transgenes in the genome of iPSCs from patients may affect their differentiation potential or induce malignant transformation, Jaenish et al. derived PD patient-iPSCs free of transgenes using Cre-recombinase to excise the reprogramming factors. These patient-iPSCs showed a global gene expression profile more closely related to hESCs and hiPSCs carrying the transgenes [101]. DA neurons from the iPSCs with the *LRRK2* mutation (G2019S) were found to be sensitive to oxidative stress and have increased expression of key oxidative stress-response genes and α- synuclein aggregation [102]. After correction of the *LRRK2* G2019S mutation in iPSCs, the degenerated DA neurons was rescued, supporting the idea that LRRK2 mutation plays important roles in the pathogenesis of PD [103]. Emborg et al. reported that autologous transplantation of rhesus monkey iPSC-derived neural progenitors to the brain of MPTP-induced hemiparkinsonian rhesus monkeys survived well and differentiate into neurons, astrocytes, and myelinating oligodendrocytes [104]. Isacson et al. used stromal feeder cell-based protocol to differentiate the virus-free PD-iPS cells into DA neurons and transplanted the DA neurons into the 6-OHDA- lesioned rats. They found that these DA neurons survived and mediated functional improvements in PD rats by reducing apomorphine-induced rotational asymmetry [98]. The same group producted the midbrain dopamine neurons derived from the cynomolgus monkey (CM) iPSC and autologously transplanted into the MPTP-lesioned CMs. They found that autologous iPSC dopamine neurons can provide long-term functional recovery and transplanted cells survive for up to 2 years and reinnervate the host brain [105]. Recently, we worked all the way from isolation of skin fibroblasts of PD patients and control individuals, to the generation of iPS cells by retrovirus-mediated expression of *OCT4, SOX2, c-MYC *and *KLF4*, to the differentiation of iPS cells to neural stem cells (NSC) and DA neurons, and finally to the transplantation of the iPS cells-derived NSCs to the striatums of the 6-OHDA-induced PD rats. We found that iPS cells carrying the transgenes can also be differentiated into the DA neurons in vitro as well as survive and be differentiated into neurons and DA neurons in PD rats. The grafted iPS cells-NSCs significantly improved the motor defects of PD rats from the 4th week to the 16th week [9]. Our results showed that iPS cells carrying the transgenes can be differentiated into DA neurons in vitro and in vivo; however, the differentiation efficiency of neurons and DA neurons need to be further improved by modifying the cell culture protocols, including growth factors and iPS cells together for transplantation, or increasing the dose of immune-suppressive agents to reduce the immune-rejection against the human-derived cells.

Research advances have been made towards improving effectiveness of iPSC generation in the absence of c-Myc. Inhibitors of DNA methyltransferase, histone deacetylase, and valproic acid (VPA) were reported to improve reprogramming efficiency, particularly improving efficiency by two orders of magnitude – up to 10 % – without induction of c-Myc. Stadtfeld et al. used non- integrating, replication-incompetent adenoviruses expressing the classic four transcripton factors to reprogram mouse liver cells into iPSCs [106]. Okita et al. developed a protocol by which repeated transfection of plasmids containing the appropriate genes (one containing the complementary DNAs of *Oct3/4*, *Sox2*, and *Klf4*; the other, *c-Myc*) into embryonic fibroblasts, resulting in iPSC cells [107]. This protocol was adapted for use in human cells by Kaji et al. [108], though the transposon piggyBac was utilized in the process, with the risk of residual sequences and chromosomal distruptions. A further approach to iPSC generation has been the use of nonintegrating episomal vectors, which allows the derivation of iPSCs that are completely free of vector and transgene sequences [109]. The DNA vector-free, direct protein transduction system was also proposed to generate iPSCs to eliminate potential risks associated with chromosomal integrations and mutations [110]. Consistent similarity in cellular and differentiation properties were found between hiPSCs from integrating and non-integrating reprogramming factors [7] . But one study showed that protein-based reprogramming of cells into hiPSCs resulted in cells that behaved most similarly to hESCs, without showing obvious exogenous reprogramming gene expression [111].

A major limitation for current ES/iPS cell differentiation protocols is the lack of clinical-grade DA neurons with a stable phenotype, the A9-subtype ventral midbrain DA neurons. To overcome this problem, Isacson et al. have developed an efficient differentiation and sorting strategy for DA neurons from both human ES/iPS cells. The NCAM (+) /CD29 (low) enriched VM DA neurons were sorted from pluripotent stem cell-differentiated neural cells. Molecular studies showed that the sorted neurons were positive for FOXA2/TH and EN1/TH and had increased expression levels of* FOXA2, LMX1A, TH, GIRK2, PITX3, EN1*, and *NURR1*, indicating that the sorted neural cells are DA neurons. After transplantation, this population of iPSC-derived DA neurons was able to restore motor function of 6-OHDA- lesioned rats. The transplanted sorted cells were found to be integrated in the rat brain tissue with TH+/hNCAM+ staining in the host striatum. This study provided experimental evidence for the feasibility and safety of iPSC-derived cell therapies in the future [99].

Another issue is the similarities and differences between iPSCs and ESCs. Several groups succeeded in generating both mouse iPSCs and human iPSCs epigenetically and developmentally identical to ESCs through improvement of end points for the reprogramming process [112, 113]. Modifications have also been made in a bid to reduce the mutagenic potential of the retroviruses and lentiviruses being used. For example, the reactivation of the *c- Myc* retrovirus particularly increases the risk of mutations, hence increasing the posibility of tumerogenicity [114]. Substituting *Nanog* and *Lin28* for *Klf4* and *c-Myc* [88] has been found to be one way to reduce this risk. However, eliminations of *c-Myc *from the protocol resulted in far lower efficiency of iPSC formation. This suggests that the role of *c-Myc* is to accelerate proliferation or otherwise enhance the speed of events establishing pluripotency, while not being necessary in the establishing of pluripotency itself [115]. Though the generated iPSCs were very similar to ESCs in morphology, growth properties, and differentiation into different germ layers, differences between ESCs and iPSCs were detected; this may be caused by using different iPSC lines [116]. In order to know the similarity and difference between iPSCs and hESCs at the molecular levels, Koyanagi-Aoi et al. analysed 49 human iPSC lines and 10 hESC lines. They found that only two iPSC lines have varied gene expression and DNA methylation. In vitro neural differentiation was compared between 40 human iPSC lines and 10 hESC lines, and only 7 iPSC lines had some undifferentiated cells after neural differentiation and formed teratoma when transplanted into mouse brains. This study indicated that iPSCs are very similar to hESCs with some difference in the specific iPSC lines [117].

Once again, it is important to note that such protocols must be adapted to a xeno-free, scalable system for the clinic. Rodriguez-Piza et al. [118] reprogrammed human fibroblasts to iPSCs under strict xeno-free conditions, providing a path to GMP applicability. Chen et al. described a suspension culture system for hESCs which was adapted by O’Brien [119] and Laslett [120] for use in hESCs and hiPSCs (reviewed by Serra et al. [121]). Such systems allowed long-term culture while retaining normal karyotype, appropriate marker expression, and pluripotency, and moreover allowed cryopreservation of cells. The future direction of pluripotent stem cell generation for clinical use lies in the use of such suspension culture bioreactors, with the added challenge of maintaining quality-control of the derived cells. To reduce the effects of transgenes on functions of the iPS cells, several labs developed methods to use two or three factors to generate iPS cells. Deng et al. reported that iPS cells can be generated from mouse embryonic and adult fibroblasts by a single factor of OCT4 in combination with small molecules of VPA, CHIR 99021, and TGF-β inhibitor [91]. A recent study indicated that the derivation of rhesus monkey naive iPSCs can be obtained with only small molecules, omitting the OCT4, providing a valuable cell source for use in preclinical research and disease modeling [122].

#### Directly induced dopamine neurons (iDA neurons)

Though iPSCs give great potentials to the cell-based therapy for PD, the complicated procedures for generation, characterization, and differentiation into the DA neurons push the researchers to find other convenient methods to obtain DA neurons. In addition, the human iPSC-derived neural stem cells or dopamine neurons may contain the undifferentiated cells which can cause tumor formation and limit their clinical application. Direct generation of iDA cells from somatic cells might have significant implications for understanding critical processes for neuronal development in vitro disease model and cell replacement therapies. One of the other approaches is to directly induce the fibroblasts of PD patients to DA neurons (iDA neurons). Recent studies have reported the success of generating DA neurons by directly reprogramming the fibroblasts with different combinations of transcription factors *Mash1 (Ascl1), Nurr1 (Nr4a2), Lmx1a, Ngn2, Sox2*, and *Pitx3* [123–125].

Caiazzo et al. first reported the production of the dopamine neurons directly reprogrammed from human and mouse fibroblasts. They identified three transcription factors, *Mash1* (*Ascl1*), *Nurr1* (Nr4a2) and *Lmx1a*, which are able to directly convert mouse and human fibroblasts to functional dopaminergic neurons. They have showed that the directly converted dopamine neurons have electro-physiological activity similar to the dopamine neurons [123]. Kim et al. reported that lentiviral expression of eight different transcription factors of *Acsl1, Mytl1, Brn2, Lmx1a, Lmx1b, Nurr1, Pitx3 *and *EN1* in mouse fibroblasts is sufficient to induce midbrain dopaminergic neurons-like cells expressing dopamine neuron marker of Pitx3. They found that two of the eight transcription factors, *Acsl1* and *Pitx3* are necessary for inducing fibroblast to dopamine neurons. Importantly these directly reprogrammed DA neurons function in mouse model of PD [126]. Kim et al. combined the transcription factors of which induced the fibroblasts to the neural progenitor cells (NPCs) and the culture environment containing SHH and FGF8 to induce the dopaminergic neuronal progenitors which can produce the dopamine neurons expressing TH and releasing dopamine [125].

Since most direct reprogramming methods are using lentiviral vectors to express the genes related to development of DA neurons, these transgenes may integrate into the genome of the iDA neurons and produce mutagenesis in iDA neurons. In addition, the fibroblasts from PD patients may also carry the genetic mutations of the genes such as *SNCA, Parkin, LRRK2, and GBA* as we discussed in iPSCs. These issues can cause safety concerns for clinical use of the directly reprogrammed DA neurons. However, the research advancement will eventually overcome these issues and bring these cells to clinical trials for PD.

### Future aspects and challenges for cell-based therapy of PD

As we discussed above, the NSCs and DA neurons from fetal brain and hESCs are not suitable for clinical use because of their immune-rejections and ethical issues. The availability of iPSCs and iDA neurons paved the road for autologous cell-based therapy of PD. A clinical trial to use iPSCs for the treatment of eye disorders has been initiated in Japan. However, several aspects of iPSCs need to be resolved before they go to clinical use. These include low yields of DA neurons, genetic and epigenetic abnormalities, and the safety of iPSC-derived cells.

#### Low yield

Though low yields of fully reprogrammed cells are a recurring problem, this is by no means an inherent property of iPSC generation, and there will continue to be yield improvements in the future. The original yields of 0.05 % have been increased by various factors, such as the addition of VPA and other chemicals in generating iPSCs [127]. Yamanaka et al. proposes the stochastic model, under which most or all differentiated cells have the potential to become iPSCs. Indeed, though iPSCs are typically generated from fibroblast cells, they have been generated from a wide array of cells from all three cell lines (mesodermal, endodermal, and ectodermal). Stadtfeld et al. [101] used liver cells; Aoi et al. [128] used stomach and liver cells; Aasen et al. [129] used human hair cells, indicating that cells can theoretically be sourced from virtually anywhere on the adult human, with varying yields across experiments. In fact, Aasen et al. found that keratinocyte-derived iPSCs from adult human hairs were indistinguishable from ESCs and generated with a 100-fold increase in efficiency compared to human fibroblast reprogramming [129]. In any case, future avenues must include comparisons between method efficiencies, with the goal of optimizing protocols for maximum cell yield of iPS cells.

#### Genetic and epigenetic abnormalities

There remain concerns regarding epigenetic memory in iPSCs and iDA neurons towards a cell fate related to their donor source and otherwise maintaining a reprogramming signature after differentiation [130, 131]. The lentivirus or retrovirus-mediated reprogramming methods should be replaced by non-integrating vectors to express the reprogramming genes or combine with small molecules for the generation of clinical applicable iPS cells [132]. Some iPSCs from PD patients may also contain gene mutations such as point mutations, chromosomal structure variations, gene duplications, and deletions in the genes of *SNCA, Parkin, LRRK2, GBA* or others [103, 133–137]. The cells derived from iPSCs with genetic mutations are not suitable for direct transplantation as the functions of cells are affected by the genetic mutations. Several protocols have been developed to correct the mutation in PD patient-derived iPSCs. It was reported that the SNCA mutation (A53T) in iPSCs could be repaired by a zinc-finger nuclease (ZFN)-mediated nuclease approach and the ability to differentiate into dopaminergic neurons was not affected by genetic correction of the A53T mutation in the patient-derived iPSCs. PCR genotyping and sequencing analysis confirmed the correctly repaired patient-derived iPSC lines [137]. A recent study showed that the LRRK2 G2019S mutation in iPSCs was corrected and the LRRK2 mutation correction produced phenotype rescue in differentiated neurons [103].

#### Safety and purity

To obtain iPSC-derived NSCs or DA neurons for the treatment of PD, it is required that the residues of undifferentiated iPSCs should be less than 1 % to avoid teratoma formation after transplantation. Approaches have been developed to sort the iPSC-derived cells with FACS or other non-invasive magnetic selection. In addition, the cell culture should be carried in feeder-free conditions to avoid the contamination of animal sources. Currently, murine-derived feeder cells are widely used to maintain hESCs and hiPSCs. Also, culture medium containing fetal bovine serum (FBS) is normally used for the culture of these feeder cells. This will cause the allogenic cell contamination of the iPSC-derived cells. A recent study developed a feeder-free system to culture the hESCs and iPSCs in the StemFit™ medium, taking a big step toward making clinically-applicable GMP-standard cells [138].

## Onwards to the clinic: conclusion

Cell replacement therapy is a promising avenue for the treatment of PD and other neurodegenerative disorders. The use of all cell sources derived – fetal NSCs, ESCs , iPSCs and iDA neurons – is fraught with ethical, logistical, and safety concerns. However, scientific research is making great progress in the development and characterization of iPSC derived cells for PD. iPSCs and their derivatives injected into animal models have shown promise in treatment of disorders such as PD; however, iPSCs have not been used in clinical trials for PD. There are some limitations/disadvantages associated with iPSCs. A relevant therapeutic progenitor or mature cell type may be identified and grafted in such treatments; in the case of PD, the options are, of course, iPSC-derived NSCs and iPSC-derived DA neurons. Theoretically, these two should act just like their non-iPSC derived counterparts –in actuality, because of the concerns mentioned above, the unique iPSC heritage of such cells sometimes poses its own unique set of problems.

Pre-clinical studies on viability might also be necessary to establish the scope of the treatment. iPSCs would not be moved to clinical trials at least until iPSCs are better understood and efficient and safe methods for reprogramming and gene correction are developed. The pace of progress will no doubt continue to speed along in the years to come, and it is therefore quite likely that within our lifetime we will witness the jump from dish to clinic.
